# Experimental Exploration of Properties and Characterization of *Nerium oleander* L. Plant Stem Fibers and Their Polymer Composites

**DOI:** 10.3390/polym18151921

**Published:** 2026-08-05

**Authors:** M. Ramesh, M. Tamil Selvan, A. Felix Sahayaraj, P. Ramya, C. Deepa, M. Sathishkumar

**Affiliations:** 1 Department of Mechanical Engineering, Sri Shanmugha College of Engineering and Technology, Salem 637304, Tamil Nadu, India; 2 Centre for Advanced Materials and Testing, Department of Mechanical Engineering, Sri Eshwar College of Engineering, Coimbatore 641202, Tamil Nadu, India; 3 Department of Mechanical Engineering, KIT-Kalaignarkarunanidhi Institute of Technology, Coimbatore 641402, Tamil Nadu, India; 4 Department of Computer Science and Engineering, Mahendra Engineering College, Namakkal 637503, Tamil Nadu, India; 5 Department of Computer Science and Engineering, Adithya Institute of Technology, Coimbatore 641107, Tamil Nadu, India; 6 Department of Mechanical Engineering, Amrita School of Engineering, Amrita Vishwa Vidyapeetham, Chennai 601103, Tamil Nadu, India

**Keywords:** *Nerium oleander* fiber, polymer composites, chemical composition, mechanical properties, thermo-gravimetry analysis, Fourier transform infra-red analysis

## Abstract

This study aimed to investigate the physicochemical properties and composites of a novel cellulosic fiber extracted from *Nerium oleander* L. plant stem. *Nerium oleander* fibers (NOFs) were separated from mature *oleander* plants using a microbial degradation technique, and their composites (NOFCs) were fabricated using a compression molding technique. The chemical composition, physical and thermal behavior of NOFs, and mechanical and water absorption properties of NOFs and NOFCs were investigated. The findings show that NOFs have a cellulose content of 57%, hemicellulose content of 13%, lignin content of 16%, density of 1.46 g/cc, and crystallinity index (CI) of 57.14%. The results further revealed that NOFs had a tensile strength of 438 MPa and a strain rate of 1.8%, whereas NOFCs had a maximum tensile strength of 58.42 MPa. NOFs can withstand temperatures up to 357 °C, according to a thermogravimetric study, and the functional groups were analyzed using Fourier-transform infrared (FTIR) spectroscopy. Scanning electron microscopy (SEM) studies showed that the surface morphology and fractured surfaces of the NOFs and their composites were smooth and circular in cross-section.

## 1. Introduction

Plant fiber-reinforced composites (PFRCs) have become a trending topic among researchers as a replacement for synthetic fiber composites owing to increased environmental awareness [1]. In the past few decades, various synthetic fibers have been employed as reinforcements in polymer matrix composites (PMCs) [2]. Synthetic fibers have some drawbacks, such as environmental pollution and non-biodegradability [3]. Plant-based fibers have been suggested as a possible alternative to man-made fibers because of their excellent features, as they are lightweight, environmentally friendly, readily available, non-abrasive on processing equipment, and corrosion-resistant [4]. PFRCs are increasingly used in industries, automobiles, electronics, and household appliances [5,6]. Many researchers have studied the mechanical and thermal properties of novel bio-based cellulose fibers extracted from various plants [7]. Fibers with high cellulose content, proper bonding, load-carrying capacity, stability against rising temperature, and light weight can feasibly substitute synthetic fibers [8]. In contrast, plants that produce such fibers grow exclusively in certain places that are dependent on environmental circumstances [9]. These advancements may result in improved environmental outcomes for items that are partially or fully biodegradable composites [10].

As the world’s population grows, so does the need for cellulose fibers in the automotive and textile industries [11]. Agro-residues have been the subject of much research on their beneficial use in various fields [12]. A large quantity of agricultural waste can be used to produce cellulose fibers and other high-value products [13]. The strength of agricultural waste fibers is primarily determined by their position within the plant [14]. The length of fibers taken from stems and leaves is longer than that of fibers derived from fruits and seeds [15]. However, due to various factors, such as low fiber yield, lack of adequate extraction tools, and poor performance compared to well-established natural fibers, only a small percentage of them may find industrial uses [16]. Furthermore, many of the fibers produced from agricultural waste are seasonal, and the market lacks a consistent and ready supply [17]. Despite these problems, research on the processing and exploitation of agricultural waste is ongoing worldwide [18]. Using agricultural waste will not only help farmers by adding value to their products, but will also protect the environment by preventing stubble burning [19].

For the fabrication of composites, it is important to investigate novel fibers with desired properties for use as reinforcements [20]. *Nerium oleander* is a large flowering plant that can grow to a height of 20–25 feet [21]. *Oleander* is a robust plant widely utilized as a decorative plant in parks, along roadsides, and in private gardens in warm subtropical climates [22]. After harvesting the flowers, a large amount of biomass is produced that must be appropriately disposed of, posing a problem for farmers [23,24]. The harvested plants are usually allowed to dry in the field for a few days. Owing to transportation costs, farmers prefer either to utilize it or leave it as is after drying. Consequently, a large volume of dried plants remains in the field, and it is common to burn them [25,26]. Although this method appears simple and inexpensive, it results in considerable air pollution in the surrounding areas. Therefore, an alternative method of upcycling agricultural waste is highly valued [27,28].

*Nerium oleander* is a medicinal plant used in Indian folk medicine. It is a potentially lethal plant in many cases, and poisoning has been reported, along with a number of suicidal cases in South Asian countries [29]. All parts of the plant are toxic and contain a variety of cardiac glycosides, including neriin, oleandrin, cardenolides, gentiobiosyl, and odoroside. This plant species also produces secondary metabolites, such as alkaloids, flavonoids, and steroids, which have pharmacological applications. Its important pharmacological activities include antibacterial, anthelmintic, anti-inflammatory, hepatoprotective, immunopotential, antipyretic, antioxidant, antifungal, anticancer, and anti-HIV activities [30]. Research on the characterization of novel cellulosic fibers adds to the knowledge on their potential use as reinforcements [31,32]. The physicochemical and dyeing properties of NOFs were investigated by Jabli et al. [33], who found that the polysaccharide content in these fibers is very close to that of wood. The anatomical structure of different fiber samples was studied by Yigit et al. [34], who found that NOFs have the highest values of lumen width and elasticity coefficients. The biosorption nature of NOFs extracted from the seeds of the plant was investigated using methylene blue, and it was found that these fibers have excellent absorption characteristics [35]. In this experimental study, chemical analysis, tensile testing, 3D profilometer study, X-ray diffraction (XRD), thermogravimetric analysis (TGA), FTIR of NOFs, water absorption nature, mechanical properties, and SEM studies of NFRCs were conducted to describe the physicochemical characteristics of these fibers and their composites.

## 2. Materials and Methods

### 2.1. Materials

Matured *Nerium Oleander* plants were harvested in Panjampatty, a village situated in the Dindigul District of Tamil Nadu, India. NOFs were extracted from the plant using a microbial degradation process. Bio-based polylactic acid (Type: PLA 2003-D) resin was purchased in the form of pellets from Nature Tech. Pvt. Ltd., Chennai, India. The acetone solution (thinner) and silica gel (releasing agent) were supplied by Ponmani & Co. (Coimbatore, India).

### 2.2. Extraction of Fiber

**Figure 1:**
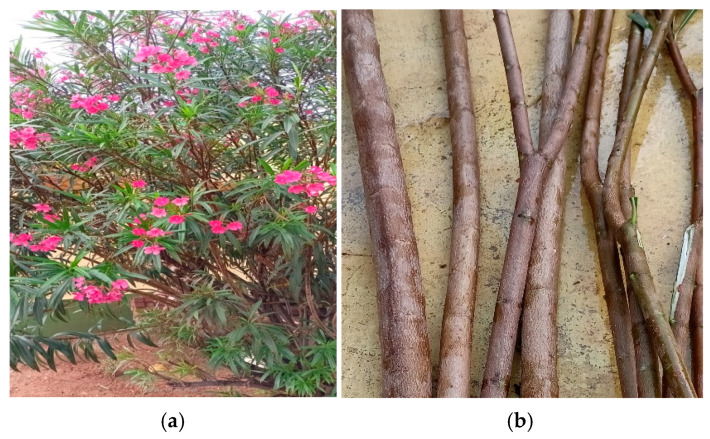

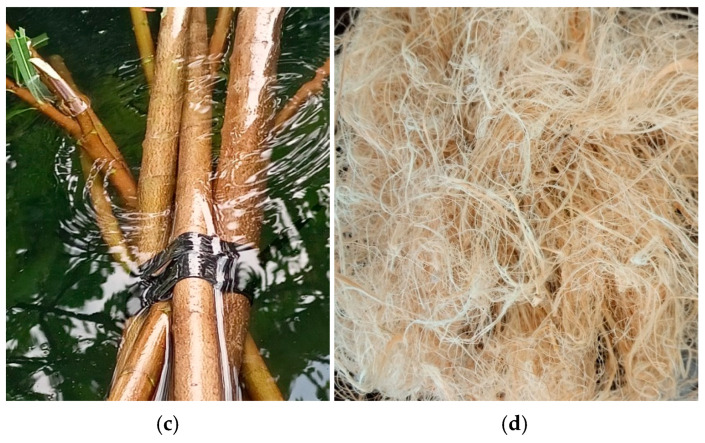
(**a**) *Nerium oleander* plant; (**b**) plant stem; (**c**) microbial degradation technique; and (**d**) extracted NOFs.

*Nerium oleander* is a small tree or shrub, generally called *oleander*, that belongs to the *Apocynaceae* family, as shown in Figure 1a. The plant stems without leaves are shown in Figure 1b. Plant stems were collected and stored in the retting tank for the microbial degradation process. The open retting tank was filled with water, and retting was performed for 15 days at a temperature of 25 °C, as shown in Figure 1c. The retted stems were removed after the prescribed time interval, and the fibers were separated from the degraded stems by hand rubbing/pulling. The fibers were then rinsed with running water and dried at room temperature. The extracted fibers are shown in Figure 1d.

### 2.3. Fabrication of Composites

NOFCs were fabricated using a compression molding method. The extracted NOFs were oven-dried at 60 °C for 24 h to remove any residual moisture. The PLA 2003D pellets were dried at 80 °C for 4 h to minimize hydrolytic degradation during processing. The dried NOFs were cut into 300 mm lengths and uniformly arranged inside a stainless-steel mold. PLA pellets were evenly distributed between successive fiber layers to obtain a fiber-to-matrix weight ratio of 1:1 (50 wt.% NOFs and 50 wt.% PLA). Before lay-up, the mold surfaces were thoroughly cleaned using acetone and coated with a silicone-based mold-release agent to facilitate easy demolding. The assembled mold was placed in a heated compression molding press and processed at 180 °C under a pressure of 5 MPa for 10 min, allowing the molten PLA to thoroughly impregnate the fiber network. After the holding period, the laminate was cooled under the same pressure to room temperature to minimize the residual stresses and prevent warpage. Finally, the edges were filed using a wood rough file, and the size of the laminate obtained was 280 × 280 × 3 mm.

## 3. Experimental Methods

### 3.1. Chemical Analysis

Standard testing procedures were used to determine the chemical composition, including cellulose, hemicellulose, lignin, wax, ash content, and moisture content, of NOFs. The wt.% of cellulose was measured using Kushner and Hoffer methods. The hemicellulose and lignin contents were calculated according to the NFT 120-008 and APPITA P11 s-78 standards. The ash content in the fibers was determined using the ASTM E1755-61 standard. The Conrad technique was used to calculate the wax, and the Sartorius model was used to measure the moisture content present in the fibers. Five samples were collected from each case to determine the average cellulose, hemicellulose, and lignin content in the fiber.

### 3.2. Density Measurement

The density of the NOFs was determined using a pycnometer. In this experimental study, the diameter of each sample (samples in total) was measured four times randomly using an optical microscope (Model: Carl Zeiss, Oberkochen, Germany), and the mean diameter was reported. A toluene solution with a density of approximately 860 kg/m^3^ was used as a solvent owing to its low carcinogenicity, and it is commonly used as a solvent in the density measurement of NOFs.

### 3.3. Single Fiber Tensile Test

Single-fiber tensile tests were conducted using a universal testing machine (UTM) following the ASTM D3379 standard. For this test, 25 fiber samples were taken, and the test was conducted at a cross-head speed of 1 mm/min, with a gauge length of 50 mm and a load cell with a capacity of 50 kN. The average tensile strength and strain rate were calculated from these results. The microfibril angle of the NOFs was calculated using the following formula:

(1) $$\varepsilon =\ln\left(1+\frac{\Delta L}{Lo}\right)=-ln(\cos\alpha )$$

where ε is the strain, α is the microfibril angle (deg.), *Lo* is the gauge length (mm), and Δ*L* is the elongation at break (mm).

### 3.4. Fiber Surface Roughness Test

In this experiment, a computerized 3D optical profilometer (Model: Zeta-20, Zeta Instruments, Milpitas, CA, USA) was used to measure the fiber surface roughness. The equipment was operated for 200 steps, with a step size of 1.082 μm. The images obtained from the Zeta-20 were used to analyze the surface features of the NOFs.

### 3.5. Mechanical Strength of NOFCs

In this experiment, the mechanical properties of NOFCs, such as the tensile strength, flexural strength, and impact strength of NOFCs, were evaluated. The tensile test was conducted using a UTM (Type: INSTRON 3369) conforming to the ASTM D3039 standard with a cross-head speed of 5 mm/min. The tensile test specimens were prepared with dimensions of 55 mm × 20 mm × 4 mm. The flexural test was carried out in the same UTM as per the ASTM D7264 standard at a cross-head speed of 10 mm/min. Specimens with a length of 150 mm, width of 15 mm, and thickness of 4 mm were used. An impact tester (Type: Izod; Make: Krystal Elmec (Kabnur, India)) was used to perform the impact test according to the ASTM D256 standard. Samples with dimensions of 65 mm for length, 15 mm for width, and 4 mm for thickness were prepared for the impact test. Five samples were tested in each case, and the average values were used for the analysis.

### 3.6. Water Absorption Studies

To examine the water absorption behavior, the NOFs and composite samples were dried for 2 h in an air-circulated oven, and the weights of the samples were measured. The water absorption test was conducted in accordance with ASTM D570 standards, and the samples were immersed in water for 72 h. The samples were then soaked in fresh water at ambient temperature. After some time, the samples were removed, wiped with a cotton cloth, and weighed again to determine the absorption level. The following formula was used to compute the percentage of water absorption:

(2) $$\mathrm{Water}\mathrm{absorption}\left(\%\right)=\frac{Wt-Wo}{Wt}*100$$

where *Wt* and *Wo* are the weights of the sample after and before immersion, respectively.

### 3.7. TGA

TGA was performed to assess the thermal degradation behavior of NOF with respect to time in a controlled nitrogen environment [36,37]. In this experiment, TGA was performed using an EXSTAR/6300 setup (Hitachi High-Tech Corporation, Tokyo, Japan) with nitrogen gas purge control. The experiment was conducted in a predetermined temperature range using alumina with a lid crucible. Approximately 2.365 mg of the fiber sample was placed in an alumina crucible. An empty alumina pan was used as a reference. The analysis was carried out under a nitrogen atmosphere over a temperature range of 31.07–987.39 °C at a constant heating rate of 15 °C min^−1^. The TG and DTA curves were recorded simultaneously to investigate the thermal degradation behavior and phase transitions of the fibers.

### 3.8. FTIR Analysis

In this experiment, the FTIR spectrum was recorded using an IR spectrometer (Shimadzu MIRacle10, Shimadzu, Kyoto, Japan). Using a ball milling machine, the NOFs were converted to a fine powder, which was combined with KBr, compressed, and pelletized. FTIR data were collected at a scan rate of 32 scans/min, a resolution of 2 cm^−1^, and a spectrum range of 4000 to 400 cm^−1^.

### 3.9. XRD Analysis

XRD is a research method primarily used to determine the phase of a crystalline substance. In this experimental study, an X’Pert-Pro PANalytical diffractometer (Malvern Panalytical B.V., Almelo, The Netherlands) was used to perform the XRD analysis of the NOFs. The Segal empirical equation was used to calculate the CI.

(3) $$CI=\frac{H22.11-H15.40}{H22.11}$$

where *H*_22.11_ is the maximum intensity of the crystalline peak and *H*_15.40_ is the intensity of the valley between the peaks. The crystallite size (CS) was calculated using the diffraction pattern of cellulose lattice planes. The CS was estimated using Scherrer’s formula.

(4) $$CS=\frac{K\lambda }{\beta COS\theta }$$

K = 0.89 is Scherrer’s constant, β is the full width at half-maximum of the peak, λ is the wavelength of the radiation, and θ is the Bragg angle.

### 3.10. SEM Analysis

The surface morphology of the fibers and fractured surfaces of the NOFCs was analyzed using a scanning electron microscope (Model: ZEISS, Oberkochen, Germany). The samples were sputter-coated with gold and then analyzed using a microscope. The equipment was operated at an accelerating voltage of 10.0 kV in a vacuum. This was done to avoid the accumulation of electrical charges on the samples during the time of scanning with an electron beam [35]. The images were captured at different magnifications.

## 4. Results and Discussion

### 4.1. Chemical Analysis

**Figure 2:**
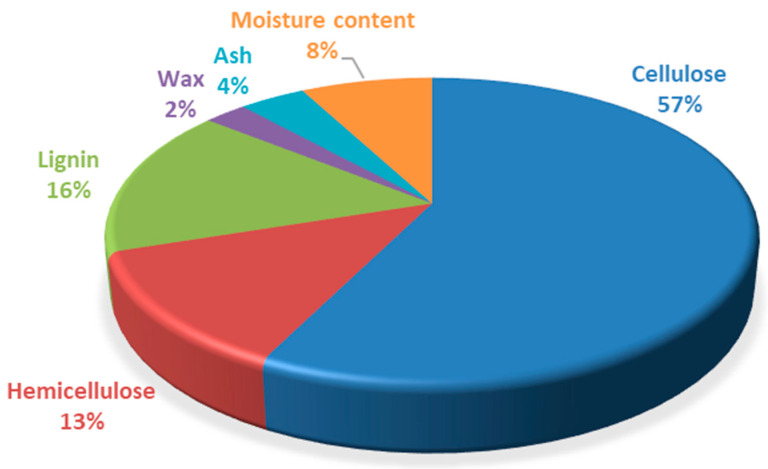
Chemical components of NOFs.

The chemical analysis of the NOFs is shown in Figure 2. The results, portrayed as a pie chart, show that the NOFs contain 57 wt.% cellulose %, 13 wt.% hemicellulose, 16 wt.% lignin, 2 wt.% wax, 4 wt.% ash, and 8 wt.% moisture content. Compared with recently reported lignocellulosic fibers, the cellulose content of NOFs is comparable to that of water hyacinth (58 wt.%) [38], *Dombeya buettneri* (58.45 wt.%) [39], *Annona reticulata* (56.26 wt.%) [40], and Fique (53–70 wt.%) [41], but lower than high-cellulose fibers such as flax (71 wt.%) [42,43], pineapple leaf fiber (70–80 wt.%) [44], *Alpinia galanga* (70.55 wt.%) [45], and *Hibiscus canescens* (68.46 wt.%) [40,43]. Conversely, NOFs exhibited a higher cellulose content than *Calathea lutea* (37.07 wt. %) [46] and *Mimusops elengi* (45.44 wt.%) [43]. Likewise, the lignin content (16 wt.%) is intermediate, exceeding that of jute (11.8–13 wt.%) [43] and water hyacinth (12 wt.%) [38], while remaining substantially lower than *Borassus aethiopum* (36.1 wt.%) [47]. Among the reported fibers, *Dombeya buettneri* exhibits an overall chemical composition closest to that of NOFs. It has similar cellulose, hemicellulose, lignin, ash, and moisture contents, suggesting a comparable reinforcement potential for polymer composite applications.

The differences in the chemical composition of these fibers are primarily due to the plant species, botanical family, fiber maturity, anatomical origin, geographical location, climatic conditions, soil characteristics, extraction method, and retting process [38,40]. Bast fibers, such as flax and jute, generally possess higher cellulose content because their thicker S2 secondary cell walls contain densely packed cellulose microfibrils that provide axial structural support [40,45]. Fibers extracted from leaves, fruits, or aquatic plants often contain greater proportions of hemicellulose and lignin to enhance their flexibility, moisture regulation, and biological adaptation [42,43]. Furthermore, extraction and retting conditions strongly influence the removal of pectin, waxes, and other non-cellulosic constituents, thereby modifying cellulose content and fiber purity [38,40]. Because the NOFs investigated in this study were produced through controlled microbial retting, the resulting composition reflects the effective removal of surface impurities while preserving the structural cellulose framework, leading to a balanced crystalline–amorphous architecture.

### 4.2. Density Measurement Analysis

In order to evaluate the density of the material, the weight of the material is considered to be directly proportional to the density of the fiber. The results indicate that the NOFs possess a density of 1.46 g/cc at room temperature. This value falls within the upper range reported for lignocellulosic fibers and indicates a compact fiber structure with adequate cell wall development.

The measured density of NOFs is comparable to that of *Mimusops elengi* (1.43 g/cm^3^) [43], *Hibiscus canescens* (1.425 g/cm^3^) [40,43], and alkali-treated *Dombeya buettneri* (1.40–1.41 g/cm^3^) [39], while being slightly higher than that of *Annona reticulata* (1.33 g/cm^3^) [40] and 2 h alkali-treated Borassus husk fiber-reinforced epoxy (1.26 g/cm^3^) [47]. Considerably lower densities have been reported for *Ficus benjamina* (0.886 g/cm^3^) [48] and *Tinospora cordifolia* (0.80 g/cm^3^) [49]. Furthermore, the density of NOFs closely matches that of conventional jute fibers (1.30–1.49 g/cm^3^), suggesting that NOFs possess a similar structural compactness and reinforcement capability that is suitable for polymer composite applications [42]. Environmental factors, such as geographical location, soil characteristics, climatic conditions, and harvesting maturity, further influence cell wall development and biomass accumulation. Moreover, retting and chemical treatments modify the fiber microstructure by removing non-cellulosic constituents, such as hemicellulose, lignin, waxes, and extractives. This results in more compact fiber architecture and a slight increase in density. The relatively high density of NOFs is attributed to their well-developed cell wall structure, balanced lignocellulosic composition, and effective removal of surface impurities during fiber extraction.

### 4.3. Single Fiber Tensile Test Analysis

The tensile strength of the fiber was affected by the high cellulose content and low microfibril angle. The results revealed that the NOFs exhibited a notable tensile strength of 438 MPa and a strain rate of 1.87%. The microfibril angle of the NOFs was estimated using Equation (1), and the value was 11.27°. The measured strength (438 MPa) closely matches those of *Mimusops elengi* (425 MPa) [43], *Ficus religiosa* (433 MPa) [50], *Ricinus communis* (428 MPa) [51], and conventional jute fibers (465 MPa) [42]. *Annona reticulata* (327 MPa) [40], *Trachelospermum jasminoides* (404 MPa) [50], and *Hibiscus canescens* (395 MPa) [40,43] showed lower single-fiber tensile strengths. Banana fibers exhibit higher tensile strengths (529–759 MPa); they also possess higher cellulose contents and optimized structural organization [42]. Among the reported fibers, *Mimusops elengi* exhibits the closest overall mechanical behavior to NOFs owing to its similar tensile strength and low elongation characteristics. The tensile response of jute is largely attributed to its lower microfibril angle (8°) and higher cellulose content (61–71%). These comparisons indicate that NOFs possess mechanical properties comparable to those of established structural natural fibers employed in polymer composite applications. The relatively low microfibril angle (11.27°) aligned the cellulose microfibrils nearly parallel to the loading direction. This improves stress transfer and axial stiffness during tensile loading. The well-developed S2 secondary cell wall contains densely packed cellulose microfibrils. It provides a primary load-bearing framework responsible for fiber strength. The balanced lignocellulosic composition, reduced lumen fraction, and minimal structural defects further enhance the mechanical performance by minimizing the stress concentration [52,53,54].

### 4.4. Surface Roughness Analysis of NOFs

The surface skewness and roughness of the NOFs were observed using a 3D optical profilometer. The images were extracted at approximately 15 μm, and the *z*-axis of the image ranged from 215 μm, with a step size in the image of 1.082 μm. The 3D profilometer results for the NOFs are presented in Table 1.

The notations Ra, Rq, and Rp describe the magnitude of the roughness. Rsk represents the surface skewness, and Rku is a kurtosis value that describes the formation of the sample height data. The distribution of peaks and valleys was measured using the skewness value of the profilometer. Based on the value, it was divided into two categories: symmetry (Rsk = 0) and asymmetry (Rsk ≠ 0) of the distribution of heights. If the Rsk value is positive, then the distribution of peaks is above the average, and the opposite sign indicates that the peaks are below the average values. The table shows that the mean Rsk value is 0.4824, which means that the distribution is neither symmetrical nor negatively skewed. Recent studies have similarly demonstrated that surface roughness is a critical parameter governing the reinforcement capabilities of natural fibers. *Albizia julibrissin* fiber exhibited an Ra of 2.759 μm [55], which is numerically close to the present NOF value (2.709 μm), indicating a comparable microscale surface roughness. Atomic force microscopy (AFM)-based studies have reported much lower roughness values because of their nanometer-scale resolution, including *Ficus tsjahela* (Ra = 35.234 nm, Rq = 43.657 nm, Rz = 171.886 nm, Rsk = −0.541, Rku = 2.515) [56], *Ficus retusa* (Ra = 39.4 nm, Rsk = −0.98, Rku = 4.20) [57], *Vitex negundo* (Ra = 16.926 nm, Rq = 29.203 nm, Rz = 194.470 nm, Rsk = −0.797, Rku = 15.342) [58], *Calathea lutea* (Ra = 3.34 nm, Rq = 5.85 nm, Rsk = −4.15) [46,59], *Guettarda speciosa* (Ra = 19 nm, Rq = 26 nm, Rz = 95 nm, Rsk = −0.895, Rku = 3.514) [60], and *Hibiscus canescens* (Ra = 7.2 nm) [40,43,56]. Furthermore, *Trianthema portulacastrum* exhibited (Rsk = 1.815 and Rku = 3.85) [61], indicating a peak-dominated and leptokurtic surface, similar to the positive skewness observed for NOFs.

**Figure 3:**
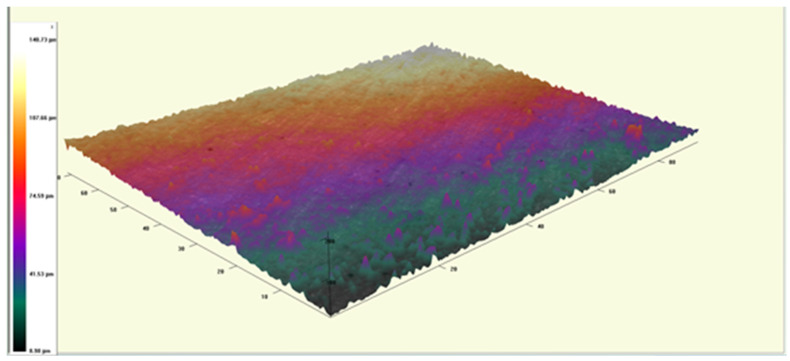
3D optical interferometry image of the NOF.

A 3D optical interferometry image of the NOF is shown in Figure 3. The field view was measured as 95 × 71 μm. For the cursor sides of both left and right, the step, distance, and angles of the values are represented as having minima of 33.39, 83.55 and 21.79; maxima of 37.56, 83.55 and 24.21; and mean values of 34.91, 83.55 and 22.57, with the standard deviations of 1.882, 0 and 1.093 and variance of 5.39%, 0% and 4.82%. The extraction of the image is represented in a variety of colors and ranges, which show the height. The position and value of the 3D profilometer were 89.391 and 11,820.000, respectively, in the graph.

**Figure 4:**
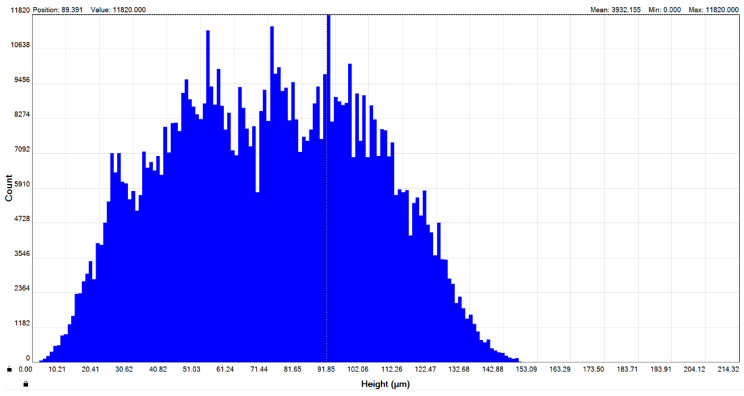
Histogram results of NOFs.

The histogram of the NOFs is shown in Figure 4. The number of repetitions or frequency of the different peaks is given on the vertical axis, and the height of the peak is given on the horizontal axis. The positively skewed histogram shows that the peak values are above the average value of the peak. This significant Ra value emphasizes the assurance of fiber–matrix bonding because the volatility in the peaks supports matrix reinforcement with the fiber. The rough surface morphology of NOFs is proportional to the influence of fiber anatomy, cellulose microfibrils, lumen morphology, and the retting process during fiber extraction. The removal of pectin, waxes, hemicellulose, and portions of lignin exposes cellulose-rich fibrillar structures. It generates surface grooves, ridges, and asperities that increase the effective surface area for the bonding. Similar observations have been reported for *Plumeria pudica* [62], *Hibiscus rosa-sinensis* [6], *Cryptostegia grandiflora* [63], *Tabernaemontana divaricate* [64], and *Tecoma stans* [65]. Rough and irregular fiber surfaces were identified as favorable for composite reinforcement because they promote mechanical interlocking and improve interfacial adhesion.

### 4.5. Analysis of Mechanical Strength of NOFCs

**Figure 5:**
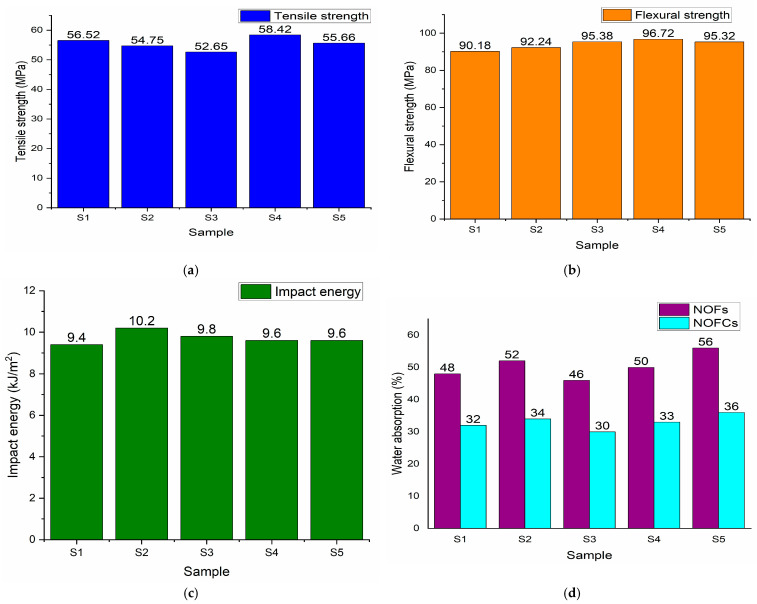
Mechanical strengths: (**a**) tensile strength, (**b**) flexural strength, (**c**) impact energy and (**d**) water absorption nature of NOFs and NOFCs.

The mechanical strengths of the NOFCs are listed in Table 2, and Figure 5a–c show the tensile and flexural strengths and impact energy-absorbing capacity of the NOFCs. Because of the high tensile strength of the NOFs, more stress transfer occurred in the inner structure of the NOFCs when the load was applied. This led to a higher tensile strength in the resulting material (58.42 MPa). Similar tensile strengths have been reported for pineapple leaf fiber/polypropylene composites (58.2 MPa) [66] and flax/waste textile fiber-reinforced PLA composites (56.27 MPa) [67], whereas sisal/nettle hybrid epoxy composites exhibited comparatively higher tensile strength (67.53 MPa) [68] owing to the hybrid reinforcement and optimized fiber architecture. Conversely, lower tensile strengths were reported for vetiver/PLA (44.64 MPa) [69] and mango seed shell/epoxy composites (29.35 MPa) [70]. This indicates that fiber morphology, interfacial adhesion, fiber loading, and matrix material significantly influence the tensile behavior of natural fiber composites [71].

The maximum flexural strength of 96.72 MPa, as shown in the figure, is due to the stress transfer that occurred at the extreme surfaces of the NOFCs when a load was applied in the transverse direction. Comparable flexural strengths have been reported for flax/waste textile fiber–PLA composites (100.32 MPa) [67] and alkali-treated Borassus husk/epoxy composites (107.67 MPa) [47], while substantially higher values have been achieved in sisal/nettle epoxy composites (162 MPa) [68] owing to optimized hybridization and reinforcement. Vetiver/PLA composites (55.29 MPa) [69] and mango seed shell/epoxy composites (48.13 MPa) [70] exhibited considerably lower flexural performances.

The figure further shows that the maximum impact energy-absorbing capacity of the NOFCs is 10.2 kJ/m^2^. Comparable impact strengths have been reported for vetiver/PLA composites (7.08 kJ/m^2^) [69], banana/sisal/PLA composites (8.00 kJ/m^2^) [72], basalt/Cissus/PLA hybrids (8.40 kJ/m^2^) [72], and kenaf/PLA composites (8.2–10.8 kJ/m^2^) [73]. However, a higher impact resistance has been achieved in pineapple leaf fiber/polypropylene composites (46.3 kJ/m^2^) [66] and sisal/PLA composites (106.0 kJ/m^2^) [74] through optimized fiber architecture, higher reinforcement content, and improved interfacial design. The results indicate that structural integrity plays a major role in the absorption of impact energy owing to the reinforcement of NOFs.

### 4.6. Water Absorption Studies

The water absorption behaviors of the NOFs and their corresponding PLA composites (NOFCs) are presented in Figure 5d and Table 2. The NOFs absorbed more water than the NOFCs. The average water absorption of the fibers was 50.4%, whereas the composites showed an average value of 33.0%, which is approximately 34.5% lower. This result indicates that the PLA matrix reduced moisture uptake by surrounding the fibers and limiting their direct contact with water. Natural fibers are hydrophilic because they contain cellulose and hemicellulose, which have numerous hydroxyl (–OH) groups. These groups easily attract water molecules via hydrogen bonding. In addition, the lumen and porous structure of the fibers allow water to move into their interiors. Similar behavior has been reported for other PLA-based natural fiber composites. Kenaf/PLA composites showed approximately 17% water absorption after 168 h of immersion [75], whereas hemp/PLA, jute/PLA, and jute/hemp hybrid PLA composites absorbed 12.5%, 10.2%, and 9.73%, respectively, after 24 h [8]. Lower values have also been reported for flax/PLA, sisal/PLA, and modal fiber/PLA composites [76].

The lower water absorption of the NOFCs was primarily attributed to the barrier effect of the PLA matrix. The matrix covers the fiber surface, reduces the number of exposed hydroxyl groups, and slows the movement of water into the composite [77,78]. However, a small amount of moisture can still enter through microvoids, fiber ends, and the fiber–matrix interface [47,79]. As water enters the composite, the fibers swell, which may weaken the fiber–matrix bonding and create small cracks [80,81]. These changes can reduce the mechanical performance of composites during long-term exposure to moisture.

### 4.7. TGA Results Analysis

**Figure 6:**
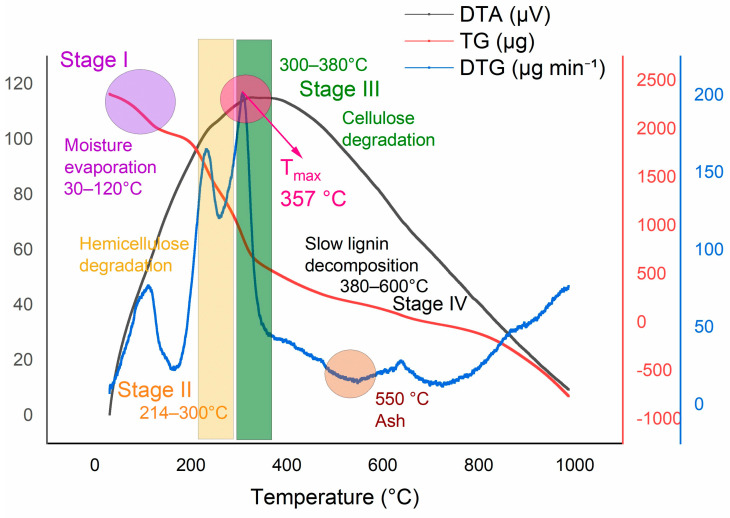
TGA thermogram of NOFs.

The thermal decomposition of the NOFs was assessed using TGA. The thermograms of the NOFs are shown in Figure 6. At 91.7 °C, the initial mass loss occurred owing to the evaporation of moisture from the NOFs. Similar moisture loss below 100 °C has also been reported for *Corypha taliera* [82], *Borassus* husk [47], jute [66,83], hemp [8,84], and Himalayan nettle fibers [85], indicating that they are hygroscopic, as cellulose and hemicellulose contain hydroxyl (–OH) groups that readily absorb water from the surrounding atmosphere [60,86]. The thermal decomposition of hemicellulose was associated with a moderate thermal peak at 214 °C with a weight loss of 12.29%. Hemicellulose has a branched and amorphous structure, making it less thermally stable than cellulose is. The degradation temperature obtained in the present study is close to the values reported for *Borassus* husk (200–300 °C) [47], *Corypha taliera* (200–300 °C) [82], and jute/coir/PALF hybrid composite (216.2 °C) [87]. The thermal breakdown of cellulose resulted in a significant peak at approximately 357 °C, with a weight loss of approximately 34.49%. The degradation temperature is comparable to those reported for *Corypha taliera* (300–400 °C) [82], *Borassus* husk (300–467 °C) [47], Himalayan nettle (300–400 °C) [85], and jute/hemp fibers (275–350 °C) [66,88]. The relatively high degradation temperature indicates that the NOFs possess good thermal stabilities. This is attributed to their cellulose-rich structure and crystalline regions that require higher energy for thermal decomposition. Above 550 °C, ash is formed owing to the strong lignin bond formation. Similar high-temperature degradation behavior has been reported for *Borassus* husk [47], vetiver [69], and sugarcane bagasse [89]. Lignin contributes to char and ash formation during the final stages of thermal degradation.

### 4.8. FTIR Analysis

**Figure 7:**
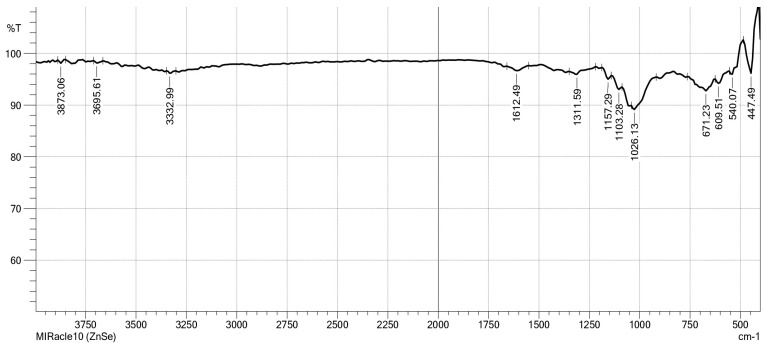
FTIR analysis of NOFs.

An FTIR graph for the NOFs is shown in Figure 7, where peaks are denoted by wave number (cm^−1^) with respect to the transmittance (%). In the single-bond region (2500–4000 cm^−1^), peak positions were found at 3873 cm^−1^, which were assigned to the hydroxy functional group of H-bonded -OH stretching in the range of 3873 to 3700 cm^−1^. Similar O–H stretching bands have been reported for *Dioscorea alata* stem fiber (3813.2 cm^−1^) [90], *Phyllanthus reticulatus* fiber (3781.72 cm^−1^) [91], and *Vitex negundo* fiber (3739 cm^−1^) [58], indicating the presence of cellulose and hydrogen-bonded –OH groups. The next peak in the single-bond region at a wave number of 3695 cm^−1^ represents the stretching vibration of hydroxyl (O–H) groups present in the lignocellulosic structure of the fiber. Similar peaks have been reported for *Mariscus ligularis* fibers (3695 cm^−1^), indicating free or weakly hydrogen-bonded hydroxyl groups associated with cellulose, hemicellulose, and lignin. The aliphatic C–H stretching vibrations of methylene (–CH_2_–) and methyl (–CH_3_) groups are observed in the 3000–2845 cm^−1^ region. Similar bands have been observed for *Annona reticulata* (2928 cm^−1^) [40,92], *Nymphaea rubra* (2922 cm^−1^) [93], *Vitex negundo* (2916 cm^−1^) [58], *Trianthema portulacastrum* (2913 cm^−1^) [61], *Grewia ferruginea* (2903 cm^−1^) [94], and *Dombeya buettneri* (2902–2904 cm^−1^) [39]. These bands are attributed to the symmetric and asymmetric stretching vibrations of the CH_2_ and CH_3_ groups present in cellulose and hemicellulose.

No peak positions were observed in the triple-bond region. In the double-bond region of 1320 to 1610 cm^−1^, the peak position of 1612 cm^−1^ increased. Similar peaks have been reported for *Dombeya buettneri* (1612 cm^−1^) [39], *Trianthema portulacastrum* (1613 cm^−1^) [61], *Mimusops elengi* (1614 cm^−1^) [43], *Ficus retusa* (1615 cm^−1^) [57], and *Mariscus ligularis* (1609 cm^−1^) [95]. The literature commonly attributes this region to carbonyl (C=O) stretching, aromatic C=C vibrations of lignin, C–O stretching of hemicellulose, and bending vibrations of the absorbed water. Another absorption band was found at 1311 cm^−1^ in the ranges of 1610 to 1370 cm^−1^ and 1520 to 1250 cm^−1^. Similar peaks have been reported for *Mariscus ligularis* (1319 cm^−1^) [95], *Grewia ferruginea* (1310 cm^−1^) [94], *Calathea lutea* (1316 cm^−1^) [46,59], *Guettarda speciosa* (1317 cm^−1^) [60], *Mimusops elengi* (1317 cm^−1^) [43], and *Ficus tsjahela* (1323 cm^−1^) [56]. These bands are commonly attributed to C–H bending, C–O stretching, and O–H in-plane bending vibrations associated with cellulose, hemicellulose, and lignin, respectively. The wavenumber 1103 cm^−1^ belongs to the vibrations of ether linkages within the lignocellulosic structure in the range of 1150 to 1110 cm^−1^. Similar bands have been reported for *Dombeya buettneri* (1104–1105 cm^−1^) [39], *Cynara scolymus* (1099 cm^−1^) [96], *Annona reticulata* (1155 cm^−1^) [40,92], *Nymphaea rubra* (1150 cm^−1^) [93], *Mimusops elengi* (1157 cm^−1^) [43], *Calathea lutea* (1157 cm^−1^) [46,59], Habara plant (1140 cm^−1^) [25], and *Mariscus ligularis* (1156 cm^−1^) [95]. This spectral region is attributed to C–O and C–O–C stretching vibrations, β-(1→4)-glycosidic linkages of cellulose, and ether linkages present in cellulose, hemicellulose, and lignin.

In the fingerprint region, the peak positions were observed at 1026 cm^−1^. Similar bands have been reported for *Ficus nekbudu* (1026 cm^−1^) [97], Fique (1026 cm^−1^) [98,99], *Dombeya buettneri* (1025–1029 cm^−1^) [39], *Mimusops elengi* (1027 cm^−1^) [43], *Ficus retusa* (1028 cm^−1^) [57], *Annona reticulata* (1030 cm^−1^) [40,92], *Pennisetum orientale* (1032–1033 cm^−1^) [100], *Trianthema portulacastrum* (1036 cm^−1^) [61], *Ficus tsjahela* (1021 cm^−1^) [56], A. galanga (1023–1030 cm^−1^) [45], and *Guettarda speciosa* (1043 cm^−1^) [60]. The literature assigns this spectral region to C–O and C–O–C stretching vibrations, C–OH bending, and alkoxy groups associated with cellulose, hemicellulose, and lignin, respectively. Low-frequency absorption bands were observed at 671 cm^−1^ and 609 cm^−1^. Similar bands have been reported for *Vitex negundo* (705 cm^−1^) [58], *Phyllanthus reticulatus* (622.89 cm^−1^) [91], *Cynara scolymus* (601 cm^−1^) [96], *Ficus retusa* (596 cm^−1^) [57], and *Grewia ferruginea* (647 and 579 cm^−1^) [94]. The literature assigns this spectral region to aromatic C–H out-of-plane bending, C–OH bending, β-glycosidic linkages, hydroxyl deformation, and skeletal vibrations associated with cellulose, hemicellulose, and lignin. The low-frequency absorption bands observed at 540 cm^−1^ and 447 cm^−1^ belong to the fingerprint region of the FTIR spectrum. Similar bands have been reported for *Grewia ferruginea* (579, 463, and 423 cm^−1^) [94], *Calathea lutea* (557 cm^−1^) [46,59], *Ficus retusa* (596 cm^−1^) [57], *Cynara scolymus* (601 cm^−1^) [96], and *Dombeya buettneri* (572 cm^−1^) [39]. The literature commonly attributes this spectral region to skeletal vibrations, C–OH bending, carboxyl group deformation, and fingerprint vibrations associated with cellulose, hemicellulose, and lignin, respectively. The functional groups of the NOFs are listed in Table 3.

### 4.9. XRD Analysis

**Figure 8:**
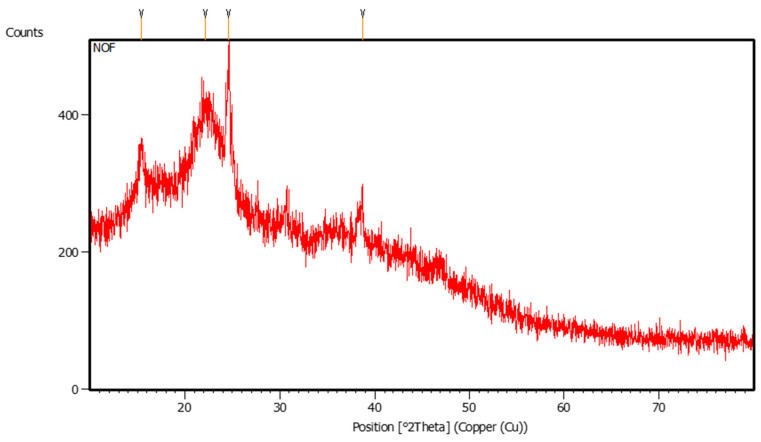
XRD analysis of NOFs.

From the XRD results shown in Figure 8, it can be interpreted that the initial peak at 2θ = 15.4097° showed a height of 72.32 cts, with a full-width half-maximum (FWHM) at 2θ = 0.4015 and d-spacing of 5.75027 Å, where lattice spacing was observed (0 1 0). Similar diffraction peaks have been reported for *Tinospora cordifolia* (15.4°) [101,102], *Vitex negundo* (15.29°) [58], *Cynara scolymus* (15.27°) [96], *Lablab purpureus* (15.52°) [103], and *Ficus tsjahela* (15.02°) [56]. The literature attributes this diffraction region to the (1–10), (110), or (101) crystal planes of native cellulose I. This reflection represents the transition between the amorphous and crystalline domains within the cellulose microfibrils and confirms the presence of an ordered cellulose framework. The second peak at 2θ = 22.1167° showed a height of 77.72 cts, with an FWHM at 2θ = 0.9368 and d-spacing of 4.01931 Å, where lattice spacing was observed (1 1 0). Similar peaks have been reported for *Vitex negundo* (21.87°) [58], *Tinospora cordifolia* (22.26°) [101,102], *Calathea lutea* (22.36°) [46,59], *Grewia ferruginea* (22.42°) [94], and *Annona reticulata* (22.60°) [40,92]. This diffraction region is widely recognized as the principal crystalline reflection of native cellulose I (cellulose Iβ), indicating highly ordered cellulose chain packing. The presence of this sharp reflection confirmed the crystalline organization of cellulose within the NOFs.

The third peak at 2θ = 24.6002°, which was the maximum peak among the diffractograms, showed a height of 185.66 cts, with an FWHM at 2θ = 0.3346 and d-spacing of 3.61887 Å, where lattice spacing was observed (2 2 0). However, fewer studies have reported a distinct reflection at this exact position. Diffraction peaks within the 22–26° region have been associated with highly ordered crystalline cellulose in several natural fibers, including *Dombeya buettneri, Cynara scolymus*, and *Trianthema portulacastrum*. The relatively high intensity and narrow peak width indicate improved crystal ordering and reduced structural disorder within the cellulose microfibrils. Such crystalline ordering generally contributes to the enhanced stiffness, dimensional stability, and mechanical performance of natural fibers. The final peak at 2θ = 38.6743° showed a height of 77.61 cts, with an FWHM at 2θ = 0.2007 and d-spacing of 2.32822 Å, where lattice spacing was observed (0 0 4). A comparable diffraction peak has been reported for *Cynara scolymus* at 38.34° [96].

Using the Segal equation and the Scherrer equation, the CI of the NOFs was calculated to be 57.14%. This value is comparable with those reported for *Aristida adscensionis* (58.9%) [104], *Guettarda speciosa* (52.99%) [60], and *Dombeya buettneri* (62.32%) [39], although lower than highly crystalline fibers such as *Nymphaea rubra* (79.44%) [93], *Lablab purpureus* (74.62%) [103], and *Phyllanthus reticulatus* (71.42%) [91]. This moderate crystallinity suggests a balanced distribution of crystalline cellulose and amorphous constituents, including hemicellulose and lignin. The CS of the NOFs was calculated to be 29.92 nm. A relatively large crystallite size indicates the presence of well-developed and ordered cellulose crystallites with fewer crystal boundaries. Previous studies have reported that *Bougainvillea glabra* (9.42 nm) [105], *Myriostachia wightiana* (3.00 nm) [106], and *Furcraea foetida* (28.36 nm) [107] contain these compounds. An increased crystallite size contributes to improved crystal stability, efficient stress transfer, and enhanced resistance to lattice deformation.

### 4.10. SEM Analysis

**Figure 9:**
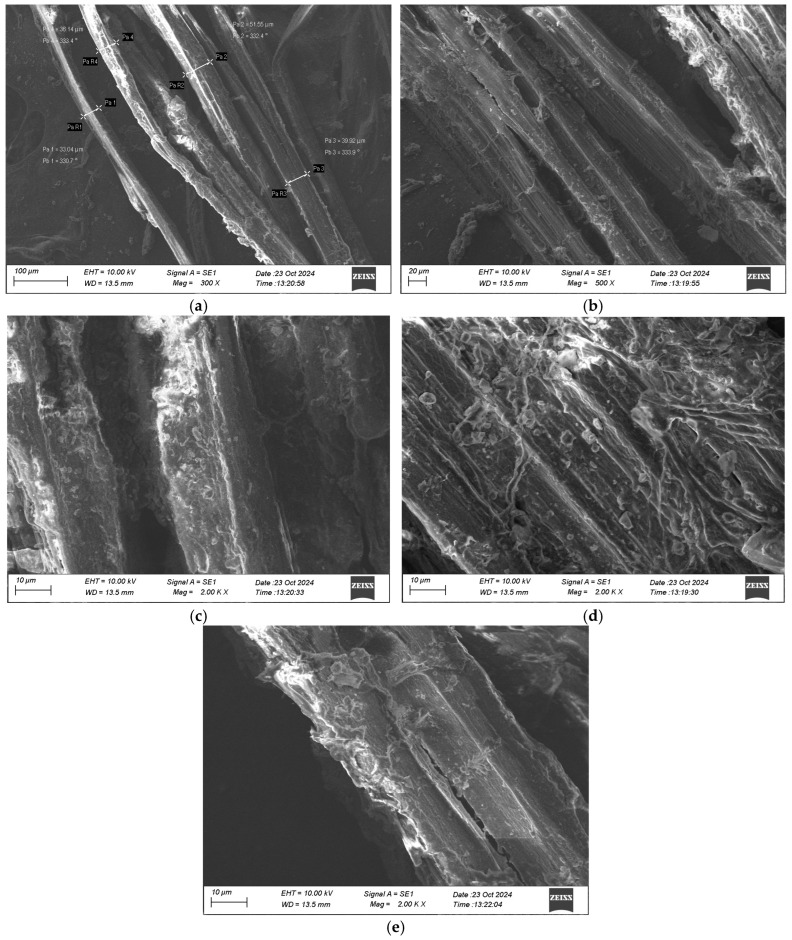
SEM images of (**a**–**c**) NOFs and (**d**,**e**) NOFCs.

The surfaces of the NOFs were examined using SEM, and the images are shown in Figure 9. Figure 9a shows a cluster of fiber images arranged randomly in the specimen holder to examine the various cross-sectional views of a fiber at different locations. A longitudinal view of a fiber is also clearly seen in this image, showing several crack-like lines across the length of the fiber that appear as long thin fibrils, possibly indicating that fiber clusters are held linked. Figure 9b shows a void in the fiber at the cross-section, which proves that the bulky cellulose content was removed from the fiber. Figure 9c reveals that lignin acts as a binder in the fiber components by forming a bridge bond with the cellulose ester. Wax and pectin are known to form protective coatings around the surface of NOFs. The flow of resin over the fiber surface and fiber bonding with the resin are clearly visible in the image presented in Figure 9d. The surface decomposition of the fiber inside the laminate owing to water absorption is clearly observed in Figure 9e.

## 5. Conclusions

In this experimental study, the physicochemical properties of NOFs, including their chemical composition, fiber density, thermal stability, tensile strength, and water absorption, were assessed. The mechanical properties and water absorption behavior of NOFCs were also investigated. The results showed that NOFs had a cellulose content of 57%, hemicellulose content of 13%, lignin content of 16%, and CI of 57.14%. It is worth noting that NOFs have a density of 1.46 g/cc, which is lower than that of other plant-based natural fibers, making them suitable for lightweight composite applications. The NOFs had a tensile strength of 438 MPa and strain rate of 1.8%, which was due to their high cellulose content and low microfibril angle. XRD peaks for these fibers were observed at 15.40°, 22.11°, 24.60°, and 38.67°, which correspond to normal cellulose I diffractions. This fiber has a CS of 29.92 nm according to previous research findings.

The absorption bands for cellulose, hemicellulose, and lignin were validated in the FTIR spectra as 3873 cm^−1^, 3695 cm^−1^, 1612 cm^−1^, and 1311 cm^−1^, respectively. The results showed that the NOFs were thermally stable up to 357 °C. Furthermore, the fiber appears to be composed of long, narrow fibrils, which could indicate that the fiber clusters are being held together. The NOFCs had a maximum tensile strength of 58.42 MPa, flexural strength of 96.72 MPa, and impact strength of 10.2 kJ/m^2^. The results showed that NOFs absorbed 1.5 times more water than NOFCs. The surfaces of the NOFs and NOFCs were observed in SEM micrographs, and surface contaminants and a non-cellulosic layer on the surface were clearly observed. This study concludes that NOFs have considerable potential for making natural fiber–polymer composites and analyzing their thermal and mechanical properties, which could lead to better product development ideas. The test results suggest that these composites are suitable for medium-load-bearing applications.

## Figures and Tables

**Table 1:** 3D profilometer results of the NOFs.

	Ra	Rq	Rpv	Rp	Rv	Rsk	Rz	Rku
**Magnitude**	2.295	3.318	25.83	14.29	11.54	0.638	16.08	5.465
	2.772	3.334	17.26	8.092	9.170	−0.318	14.36	2.390
	3.061	4.027	28.85	20.72	8.133	1.128	17.77	6.166
Min	2.295	3.138	17.26	8.092	8.133	−0.318	14.36	2.390
Max	3.061	4.027	28.85	20.72	11.54	1.128	17.77	6.166
Mean	2.709	3.499	23.98	14.37	9.613	0.4825	16.07	4.674
SD	0.315	0.3814	4.908	5.155	1.425	0.6003	1.393	1.640
Var (%)	11.6	10.9	20.5	35.9	14.8	124.4	8.67	35.1

**Table 2:** Experimental results of mechanical properties and percentage of water absorption.

Sample	Tensile Strength (MPa)	Flexural Strength (MPa)	Impact Energy (kJ/m^2^)	Water Absorption (%)	
				NOFs	NOFCs
S1	56.52	90.18	9.4	48	32
S2	54.75	92.24	10.2	52	34
S3	52.65	95.38	9.8	46	30
S4	58.42	96.72	9.6	50	33
S5	55.66	95.32	9.6	56	36

**Table 3:** Functional groups of the NOFs.

S. No.	Peak Positions (Wave Number cm^−1^)	Spectral Region	Functional Group	Major Chemical Constituent	Literature Interpretation
1	3873.06	Single-bond region	O–H stretching (hydrogen-bonded hydroxyl groups)	Cellulose	Confirms hydroxyl groups associated with cellulose and hydrogen bonding
2	3695.61	Single-bond region	O–H stretching (free/weakly hydrogen-bonded hydroxyl groups)	Cellulose, hemicellulose, lignin	Indicates free hydroxyl groups within the lignocellulosic structure
3	1612.49	Double-bond region	C=O stretching, aromatic C=C vibration, C–O stretching	Lignin, hemicellulose	Indicates carbonyl groups, aromatic lignin structure, and absorbed moisture
4	1311.59	Double-bond region	C–H bending, C–O stretching, O–H in-plane bending	Cellulose, hemicellulose, lignin	Characteristic vibration of lignocellulosic polysaccharides
5	1157.29	Fingerprint region	C–O–C stretching, CH_3_ bending	Cellulose, hemicellulose	Ether linkage and polysaccharide backbone vibration
6	1103.28	Fingerprint region	C–O and C–O–C stretching, β-(1→4)-glycosidic linkage	Cellulose, hemicellulose, lignin	Ether linkage and cellulose backbone vibration
7	1026.13	Fingerprint region	C–O stretching, C–O–C stretching, C–OH bending	Cellulose, hemicellulose, lignin	Confirms polysaccharide framework and glycosidic linkage
8	671.23	Fingerprint region	Aromatic C–H out-of-plane bending, hydroxyl deformation, skeletal vibration	Cellulose, hemicellulose, lignin	Indicates lignocellulosic skeletal framework
9	609.51	Fingerprint region	C–OH bending, β-glycosidic linkage, skeletal vibration	Cellulose, hemicellulose, lignin	Associated with cellulose backbone and hydroxyl deformation
10	540.07	Fingerprint region	Skeletal vibration, C–OH bending, carboxyl group deformation	Cellulose, hemicellulose, lignin	Characteristic fingerprint-region vibration of lignocellulosic fibers

## Data Availability

The original contributions presented in this study are included in this article. Further inquiries can be directed to the corresponding author.
